# Optimization Algorithms and Their Applications and Prospects in Manufacturing Engineering

**DOI:** 10.3390/ma17164093

**Published:** 2024-08-17

**Authors:** Juan Song, Bangfu Wang, Xiaohong Hao

**Affiliations:** 1 Department of Basic Courses, Suzhou City University, Suzhou 215104, China; haoxiaohong200866@163.com; 2 College of Mechanical Engineering, Suzhou University of Science and Technology, Suzhou 215009, China; bfwang@usts.edu.cn

**Keywords:** process parameters, optimization algorithms, response surface method, genetic algorithms, particle swarm optimization, engineering applications

## Abstract

In modern manufacturing, optimization algorithms have become a key tool for improving the efficiency and quality of machining technology. As computing technology advances and artificial intelligence evolves, these algorithms are assuming an increasingly vital role in the parameter optimization of machining processes. Currently, the development of the response surface method, genetic algorithm, Taguchi method, and particle swarm optimization algorithm is relatively mature, and their applications in process parameter optimization are quite extensive. They are increasingly used as optimization objectives for surface roughness, subsurface damage, cutting forces, and mechanical properties, both for machining and special machining. This article provides a systematic review of the application and developmental trends of optimization algorithms within the realm of practical engineering production. It delves into the classification, definition, and current state of research concerning process parameter optimization algorithms in engineering manufacturing processes, both domestically and internationally. Furthermore, it offers a detailed exploration of the specific applications of these optimization algorithms in real-world scenarios. The evolution of optimization algorithms is geared towards bolstering the competitiveness of the future manufacturing industry and fostering the advancement of manufacturing technology towards greater efficiency, sustainability, and customization.

## 1. Introduction

In modern manufacturing, optimization algorithms have become a key tool for improving processing technology efficiency and quality [1]. With the development of computing technology and the advancement of artificial intelligence, these algorithms play an increasingly important role in the parameter optimization of machining processes [2]. By precisely adjusting processing parameters, optimization algorithms can significantly improve material utilization, reduce energy consumption, shorten production cycles, and improve the quality and consistency of the final product [3]. The ultimate goal of manufacturing is to produce high-quality products with minimal cost and time consumption [4]. To achieve these goals, one of the considerations is to optimize the processing parameters.

The quality, microstructure, performance, cost, and lifespan of part machining are directly impacted by process parameters, including spindle speed, feed rate, cutting depth, tool material, and cooling conditions [5]. The determination and design of process parameters are considered the fundamental activities in the implementation of part manufacturing based on specific processes. Process parameters are influenced by process methods, component materials, and part shapes, as per the theoretical knowledge in material science and related fields [6]. For the design optimization of process parameters that are not fully determined and cannot be calculated through formulas, the “trial and error” method is predominantly utilized [7]. Optimal process parameters are selected by observing various process parameters and conducting experiments to analyze the factors influencing part processing quality. However, the complete trial and error method is characterized by a lengthy cycle, high cost, and is constrained by experimental costs and conditions. The optimization of process parameters aims at maximizing production efficiency while fulfilling product quality requirements [8].

Studying process parameter optimization algorithms for intelligent manufacturing can effectively compensate for the shortcomings of empirical or trial and error methods in the process of optimizing process parameters and improve the stability and efficiency of the production process [9]. The introduction of intelligent optimization algorithms is to assist engineers in process planning for multiple machining processes. In practical industrial applications, the manufacturing performance of parts has been improved [4]. Among the existing optimization algorithms, the genetic algorithm, simulated annealing algorithm, and particle swarm optimization algorithm are widely used in process parameter optimization problems [10]. The genetic algorithm has achieved success in many fields by simulating the process of biological evolution and searching for optimal solutions through selection, crossover, and mutation. The simulated annealing algorithm simulates the process of solid annealing by accepting better or worse solutions with a certain probability of jumping out of local extremes [11]. In addition to common intelligent optimization algorithms, deep learning algorithms have also made some breakthroughs in process parameter optimization in recent years [12]. The combination of predictive models and improved intelligent algorithms has emerged as an effective method for enhancing process optimization and machining processes. Initially, the predictive model establishes the correlation between the input and output, with the improved intelligent algorithm serving as an effective solver to obtain highly optimized solutions [4,13].

However, in practical applications, process parameter optimization algorithms also need to consider multiple aspects, such as minimizing production costs and time simultaneously, as well as maximizing profit margins [14,15]. The constraint conditions also need to meet the limitations of machine tool power, feed rate, spindle speed, and other related parameters. The use of optimization algorithms needs to consider the mutual influence between process parameters, avoiding overly single optimization of one parameter while ignoring the influence of other parameters [16,17]. With the development of artificial intelligence and deep learning technology, methods from fields such as machine learning, artificial intelligence, and expert systems can also be combined for comprehensive application in practical problems [15,18]. For example, machine learning can predict and optimize process parameters by training models and learning data, use deep learning algorithms to learn patterns and patterns from a large amount of data, and provide corresponding prediction and optimization results [19,20].

In summary, the application and development status of optimization algorithms in process parameters within the realm of engineering manufacturing are summarized in this article. The definitions, classifications, advantages, and disadvantages, as well as specific applications of optimization algorithms (response surface method, genetic algorithm, and particle swarm optimization algorithm), are analyzed. This article also looks forward to the future use of new machine learning and other optimization algorithms in parameter optimization, offering a reference for relevant engineering problems and researchers.

## 2. Classification, Advantages and Disadvantages, and Application Areas of Optimization Algorithms

Optimization of processing technology parameters is an important research direction in the manufacturing industry, aimed at improving product quality, reducing production costs, and enhancing production efficiency [21]. In the traditional process of optimizing process parameters, the most common methods are single-factor experiments, orthogonal experiments, response surface methods, and so on [22]. With the development of computational technology, the optimization methods of process parameters have also shifted from traditional empirical methods to more systematic and scientific algorithm optimizations [23,24]. The single-factor experiment and orthogonal experiment are simple and widely used, and they will not be described in detail in this section. This section mainly describes the response surface method, genetic algorithm, particle swarm optimization algorithm, and other algorithms applied to process parameter optimization, introducing their definitions, usage methods, advantages, and disadvantages.

### 2.1. Response Surface Method

**Figure 1:**
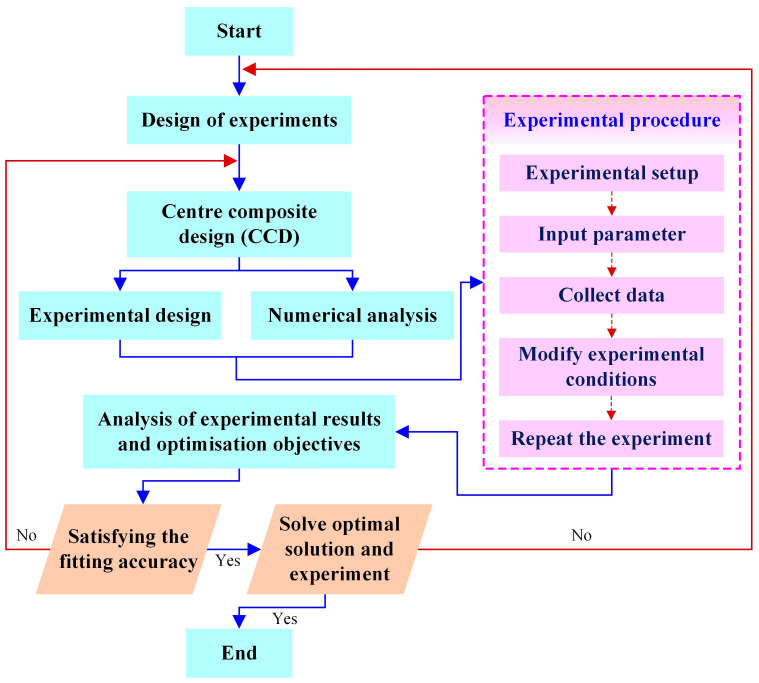
Design process of response surface method [27,28].

Response surface design is a statistical method that utilizes reasonable experimental design methods and obtains certain data through experiments [25]. It uses multiple quadratic regression equations to fit the functional relationship between factors and response values, and it analyzes the regression equations to seek the optimal process parameters and solve multivariate problems [26]. The response surface method first requires experimental design. The design process of the response surface method is shown in Figure 1 [27,28]. A suitable mathematical model is established based on a large amount of experimental data, and a graph is drawn based on this mathematical model. This method has the advantages of fewer experiments, shorter cycles, and higher accuracy [29].

The specific design process of response surface methodology can be described as follows: Firstly, a suitable experimental design (e.g., a central composite de-design) is selected to determine the range of processing parameters on the basis of a one-factor experiment, and then the response surface methodology experimental design is carried out [30]. Secondly, based on the normal distribution maps of variance, range, and residual, the response surface method experimental results are analyzed to insepct the interaction between processing parameters. Finally, the optimal solution for processing parameters is obtained.

The response surface method establishes a mathematical model between variables and response values. Based on the Taylor expansion, a polynomial fitting function can be established that only considers constant and linear terms, as shown in Equation (1) [31]:

(1) $$y=\beta_{0}+\sum_{i=1}^{k}\beta_{i}x_{i}+\varepsilon$$

where *y* is the response value in the fitting function, *β*_0_ is a constant term, *k* is the number of variables, *β_i_* is the linear impact regression coefficient of *x_i_*, *x_i_* is the input independent variable, and *ε* is the residual of the mathematical model.

In addition, a quadratic polynomial model is used to describe the main effects in process variables and the mutual effects between variables. The quadratic polynomial fitting model is shown in Equation (2):

(2) $$y=\beta_{0}+\sum_{i=1}^{m}\beta_{i}x_{i}+\sum_{i=1}^{m}\beta_{ii}x_{i}x_{i}+\sum \sum_{i<j}\beta_{ij}x_{i}x_{j}+\varepsilon$$

where *β_ii_* is the quadratic regression coefficient with respect to the independent variable *x_i_*, *β_ij_* is the linear interaction influence coefficient with respect to *x_i_* and *x_j_*, and the other terms have the same meaning as in Equation (1) above.

### 2.2. Genetic Algorithm

**Figure 2:**
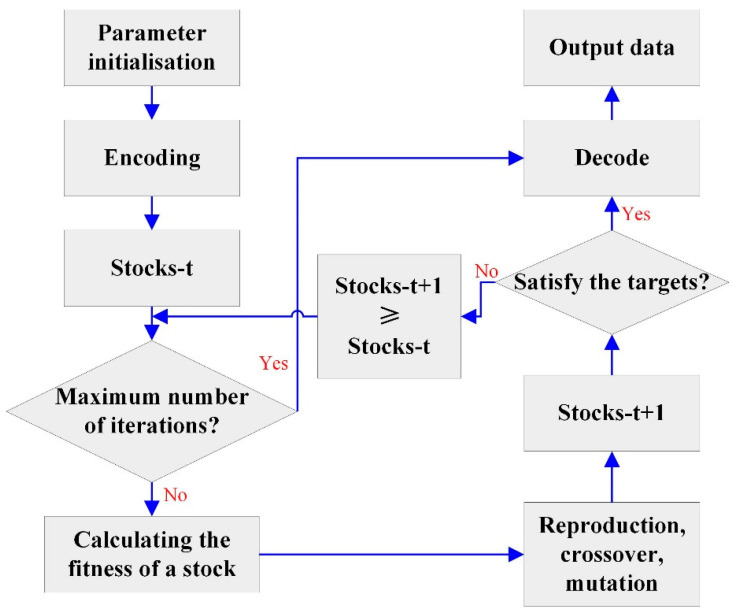
Design process of genetic algorithm.

The genetic algorithm is an optimization search method based on natural selection and genetic principles, proposed by American computer scientist John H. Holland [32,33]. The genetic algorithm is an algorithm that iteratively updates the solution of a problem by simulating the Darwinian natural selection and genetic laws in the natural evolution process of organisms in order to search for the optimal solution or approximate optimal solution [34]. The basic process of genetic algorithm is roughly as follows (as shown in Figure 2). Firstly, map the problem into a mathematical problem, that is, establish a mathematical model. Secondly, initialize a population consisting of multiple individuals, each representing a solution. Then, evaluate the individuals based on the fitness function and select excellent individuals for reproduction [35]. Subsequently, perform a crossover operation by randomly selecting two individuals for chromosome crossover, generating new offspring, and updating the optimal solution. Finally, repeat the above steps until the preset number of iterations is reached or other stopping conditions are met [36].

The standard method of genetic algorithm includes steps such as population initialization, selection, crossover (hybridization), mutation, and substitution [37,38]. The advantage of genetic algorithms lies in their adaptability and ability to handle complex nonlinear, non-convex optimization problems, and dynamic environments. In addition, genetic algorithms also have good global search ability, which can avoid becoming stuck in local optimal solutions [39]. However, the convergence speed of genetic algorithms may be slow, and it is necessary to adjust parameters such as population size, crossover probability, and mutation probability to achieve better performance.

The specific application areas of genetic algorithm mainly include function optimization, combinatorial optimization, machine learning, bioinformatics, and control signal systems [40,41]. For example, combinatorial optimization strategies, audio and image processing, signal regulation of control systems, parameters in machine learning, and protein structure prediction have broad applications and development prospects but need to solve problems such as convergence and operational efficiency [42].

NSGA-II has fast convergence and better population diversity at the Pareto front, with high algorithm accuracy. Compared to NSGA, which mainly focuses on non-dominated sorting, NSGA-II has three main running times: non-dominated sorting (constructing non-dominated sets), calculating focus distance, and constructing partial ordered sets [43]. The time complexity of NSGA is MN^3^, while the time complexity of NSGA-II is MN^2^, where M is the target number and N is the number of individuals in the population. The non-dominated sorting method is based on Pareto-dominated feasible solutions. For a typical multi-objective optimization problem, the vectors of the design variables for population individuals *i* and *j* are represented by Equation (3):

(3) $$\begin{array}{l} X^{i}=\left[x_{1}^{i},x_{2}^{i},x_{3}^{i},\cdots ,x_{m}^{i}\right]\\ X^{j}=\left[x_{1}^{j},x_{2}^{j},x_{3}^{j},\cdots ,x_{m}^{j}\right] \end{array}$$

Furthermore, the vectors of the response variables for individuals *i* and *j* in the population are represented by Equation (4):

(4) $$\begin{array}{l} R^{i}=\left[r_{1}^{i},r_{2}^{i},r_{3}^{i},\cdots ,r_{n}^{i}\right]\\ R^{j}=\left[r_{1}^{j},r_{2}^{j},r_{3}^{j},\cdots ,r_{n}^{j}\right] \end{array}$$

If any response variable in *R^i^* is not worse than the response variable in *R^j^*, and at least one response variable in *R^i^* is better than the response variable in *R^j^*, then *X^i^* dominates *X^j^*. If *X^i^* is not dominated by any other solution, *R^i^* can be represented as a point on the Pareto front, which describes the optimal solution that achieves trade-offs between optimization objectives, that is, it cannot improve any objectives without affecting other objectives [34].

Genetic algorithms can innovate crossover and mutation strategies in the future and can also break through population initialization and diversity preservation. In addition, by mixing with other optimization algorithms, genetic algorithms can be combined with other optimization algorithms (such as particle swarm optimization, simulated annealing, etc.) to utilize their respective advantages and study the convergence and parameter adaptability of genetic algorithm.

### 2.3. Particle Swarm Optimization

**Figure 3:**
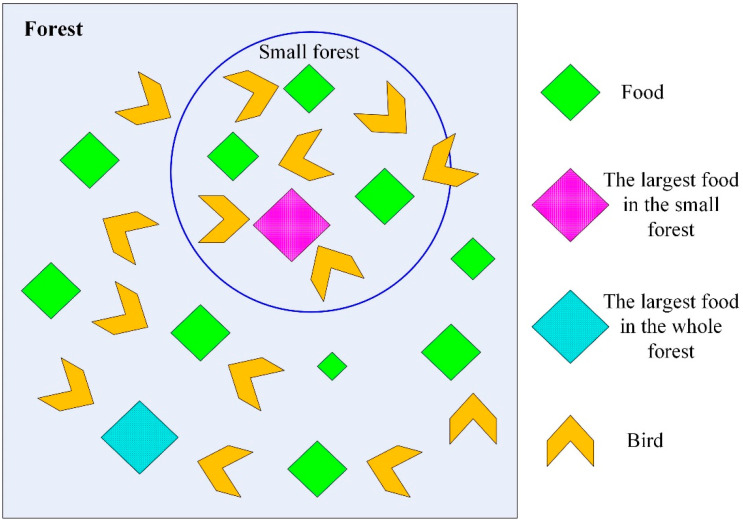
Schematic diagram of birds foraging for food.

Particle swarm optimization is an evolutionary computing technique proposed by Dr. Eberhart and Dr. Kennedy in 1995, originating from the study of foraging behavior in bird populations (as shown in Figure 3) [44]. The basic idea of this algorithm is to find the optimal solution through a collaboration and information sharing among individuals in the group.

**Figure 4:**
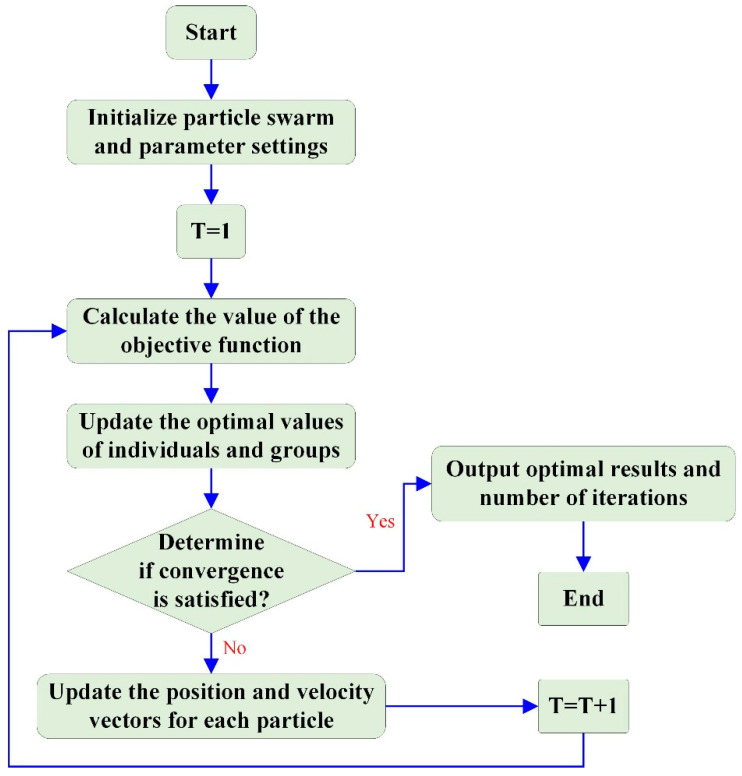
Design process of particle swarm optimization algorithm.

The particle swarm algorithm simulates birds in a bird swarm by designing a mass-free example, where particles only have two attributes, velocity and position, where velocity represents the speed of bird movement and position represents the direction of bird movement [45]. The flowchart of the particle swarm optimization algorithm is shown in Figure 4 [46].

The particle swarm optimization (PSO) algorithm proceeds through the following specific steps:

(1) Initializing the velocity and position of the particle swarm, the inertia factor acceleration constant, and the maximum number of iterations [47].
(2) Evaluating the initial fitness value of each particle and substituting it into the objective function.
(3) Using the initial fitness value as the local optimum of the current particle (dependent variable) and the position as the position of the current local optimum (independent variable).
(4) Taking the optimal local optimal value (initial fitness value) among all particles as the current global optimal value and the optimal position as the position of the global optimal value.
(5) Substituting the velocity update relation to update the flight velocity of the particles.
(6) Updating the position of each particle.
(7) To compare whether the adaptation value of each particle is better than the historical local optimum. If yes, take the current fitness value as the local optimal value of the particle and take the corresponding position as the local optimal position of the particle.
(8) To find out the global optimal value in the current particle swarm and take the corresponding position as the global optimal position.
(9) Reiterating steps 5–9 until the set minimum error or maximum number of iterations is reached. Output the optimal value and position, the local optimal value, and position of other particles [48].

In the particle swarm algorithm, particles need to update their velocity and position via Equation (5), with a specific expression, as follows [49,50]:

(5) $$v_{i}^{d}=\omega v_{i}^{d-1}+c_{1}r_{1}\left(pbest_{i}^{d}-x_{i}^{d}\right)+c_{2}r_{2}\left(gbest^{d}-x_{i}^{d}\right)$$

where *n* is the number of examples, *c*_1_ is the individual learning factor of the particle, *c*_2_ is the social learning factor of the particle, *ω* is the inertia weight of the velocity, *v_i_^d^* is the velocity of the *i*th particle at the *d*th iteration, *x_i_^d^* is the position where the *i*th particle is at the *d*th iteration, *f*(*x*) refers to the fitness at position *x*, *pbest_i_^d^* is the best position the *i*th particle has passed the *d*th iteration by, and *gbest_i_^d^* is the best position passed by all particles by the *d*th iteration [51].

Further, the position where the particle is located at step *d* + 1 is the sum of the position at step *d* and the position at step *d* multiplied by the movement time, which can be described as Equation (6) [52]:

(6) $$x_{i}^{d+1}=x_{i}^{d}+v_{i}^{d}$$

where the inertia weight *ω* decreases as the number of iterations increases, ultimately allowing the particle swarm algorithm to exhibit a local convergence ability; the expression for *ω* is shown in Equation (7) [53]:

(7) $$\omega =\omega_{\max}-\left(\omega_{\max}-\omega_{\min}\right)\frac{\mathrm{iter}}{{\mathrm{iter}}_{\max}}$$

where *ω*_max_ and *ω*_min_ are the maximum and minimum inertia weights, respectively, iter is the current iteration number, and iter_max_ is the maximum iteration number.

**Figure 5:**
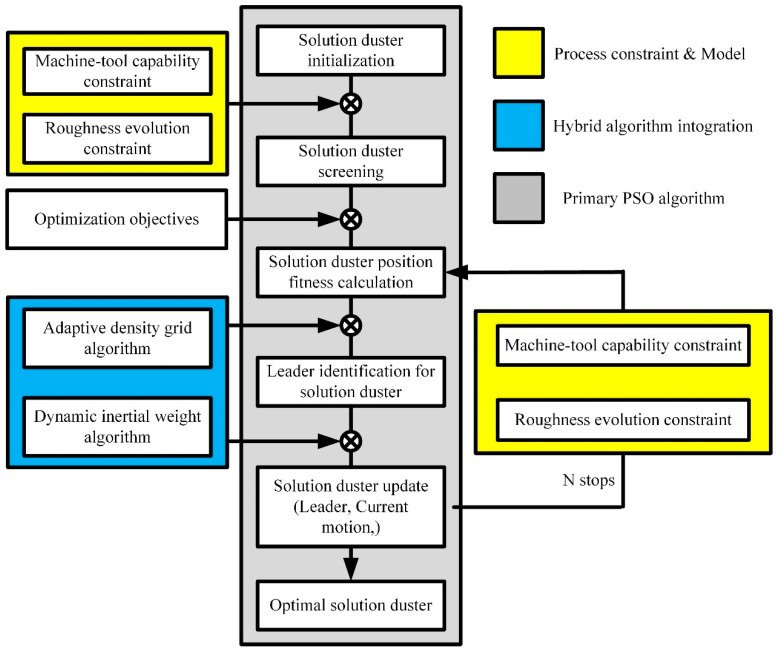
Calculation process of hybrid particle swarm optimization algorithm [4]. “Reprinted with permission from Ref. [4]. 2024, Int. J. Adv. Manuf. Technol”.

Furthermore, the above is an introduction to the basic particle swarm algorithm. With the development of technology and application needs, a hybrid particle swarm algorithm has been developed to solve related engineering problems and optimize process parameters [54,55]. As shown in Figure 5, the calculation process of the hybrid particle swarm optimization algorithm is introduced.

Firstly, during the initialization process, some machining process parameters will be selected based on the relevant performance parameters of the machine tool. Then, the appropriate fitting degree of the process parameter position will be calculated through the objective function, and the PBest solution set will be obtained. In addition, the adaptive density grid algorithm is integrated into the particle swarm algorithm to select the appropriate solution set. Subsequently, the dynamic inertia weight algorithm is used to determine the weight of the current motion’s impact on the new position [4]. Finally, after a certain number of iteration steps, the final process parameter solution can be obtained.

The particle swarm optimization algorithm can be applied to optimize the parameters of many manufacturing processes in the following ways: milling, turning, drilling, injection molding, welding process, laser cutting, laser welding, other laser processing, surface treatment, electroplating, painting, heat treatment, and 3D printing [56,57]. The application of the particle swarm optimization algorithm in these manufacturing processes usually needs to be realized by adjusting the algorithm parameters and designing a reasonable fitness function in combination with specific process characteristics and optimization objectives [58].

### 2.4. Machine Learning and Other Algorithms

Machine learning is an interdisciplinary field that covers the knowledge of probability theory, statistics, approximation theory, and complex algorithms. It uses computers as tools and is committed to simulating human learning methods in real time; moreover, it divides existing content into knowledge structures to effectively improve the learning efficiency. Optimization algorithms in machine learning are mathematical methods used to solve model parameters or features, which can improve the accuracy and generalization ability of the model. Common optimization algorithms include the decision tree algorithm, naive Bayesian algorithm, artificial neural network algorithm, and so on.

#### 2.4.1. Decision Tree Algorithm

**Figure 6:**
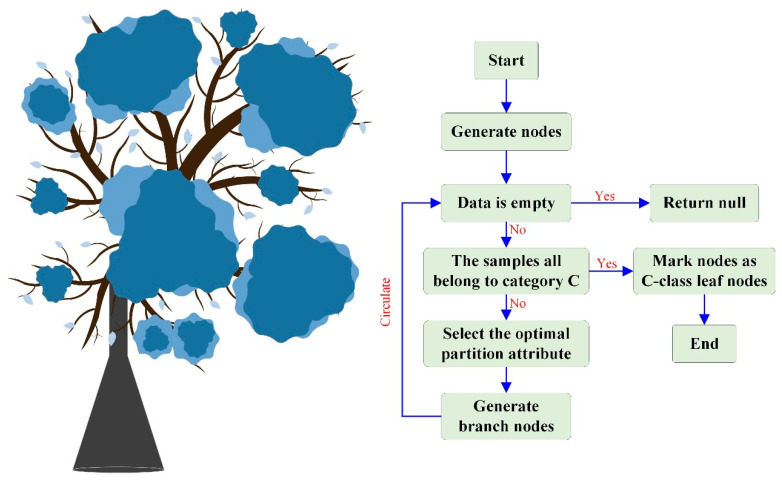
Steps in the process of generating decision trees.

Decision trees are one of the machine learning algorithms that can handle both complete and incomplete data. Decision trees and their variants are algorithms that divide the input space into different regions, each with independent parameters. The decision tree algorithm fully utilizes the tree model, as shown in Figure 6. The path from the root node to a leaf node is a classification path rule, and each leaf node represents a judgment category. First, the samples are divided into subsets; then, they are split and recursed until the same type of sample is obtained for each subset. From the root node, the test is started from the subtree and then to the leaf nodes to obtain the predicted categories. This method is characterized by a simple structure and efficient data acquisition [59].

**Figure 7:**
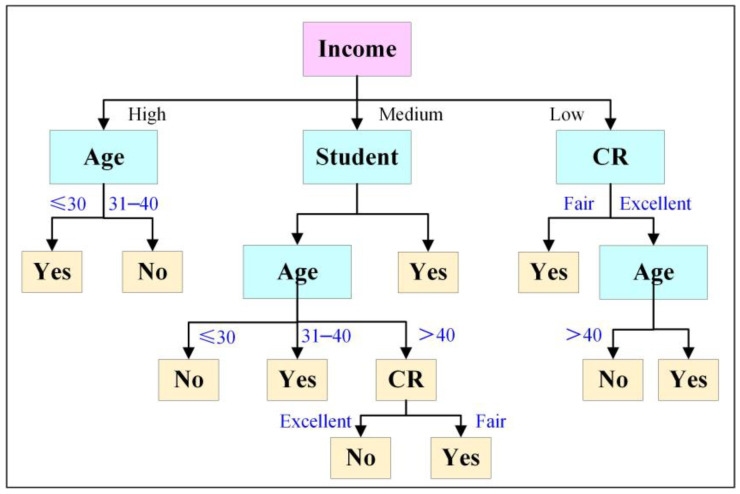
Example of the application of the decision tree algorithm [64]. “Reprinted with permission from Ref. [64]. 2024, J. Appl. Sci. Technol. Trends”.

The decision tree algorithm is widely used in fields such as machine learning, image recognition, traffic control, and biomedicine [60]. The decision tree algorithm can effectively compare numerical features and thresholds during the testing process [61,62]. The decision tree algorithm is commonly used for grouping functions, achieving precise analysis of classification category features through data mining [63]. Figure 7 shows an example of the application of the decision tree algorithm [64].

The main types of decision tree algorithms are iterative dichotomization, classification and regression trees, multivariate adaptive regression spline, conditional association trees, and so on [65,66]. In addition, the main objective of decision tree algorithms is to build a training model and then use machine learning to compute the decision rules from the training data, which in turn gives the thresholds for predicting the target variables [67,68]. However, the advantages and disadvantages of decision tree algorithms are also more significant. First, the decision tree algorithm is easy to understand and can categorize categories and numerical results [69]. Second, the decision tree algorithm does not make a priori assumptions about how good or bad the results will be [70]. However, the decision tree algorithm may make wrong decisions because the optimal decision-making mechanism is blocked. Secondly, the decision book algorithm requires a relatively large number of training samples, which increases the computational cost [71,72].

#### 2.4.2. Naive Bayes Algorithm

**Figure 8:**
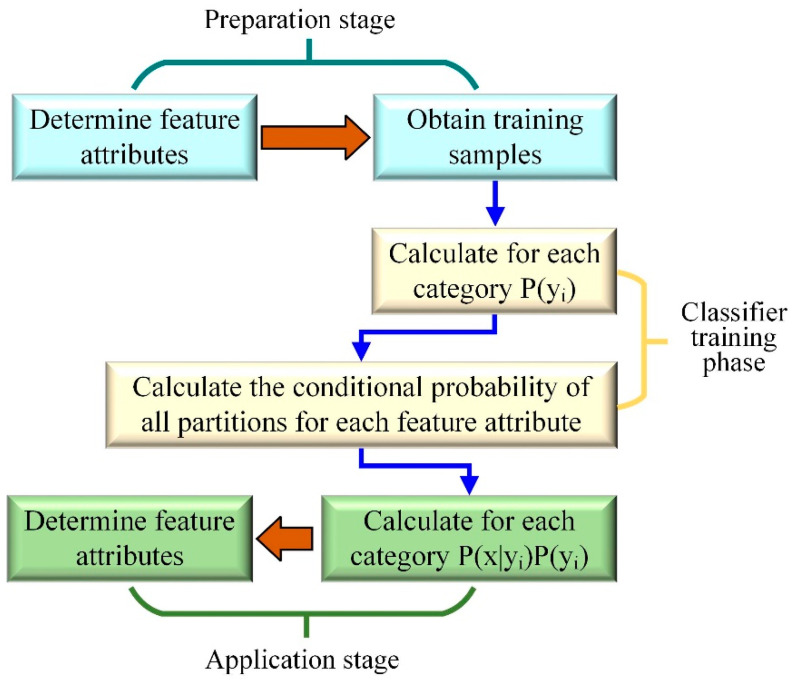
Process of naive Bayesian algorithm.

The naive Bayes algorithm is a classification method based on the Bayesian theorem and independent assumption of feature conditions. For a given training dataset, the joint probability distribution of the input and output is first learned based on the independent assumption of feature conditions. Then, based on this model, for the given input x, use the Bayesian theorem to find the output y with the highest posterior probability. The algorithm process is shown in Figure 8.

According to the description of the naive Bayesian algorithm mentioned above, it is necessary to first understand the Bayesian theorem. The Bayesian theorem refers to the probability under the condition of event *B* (occurrence), which is different from the probability of event *B* under the condition of event *A* (occurrence), but there is a definite relationship between the two. The Bayesian theorem is a description of these two relationships, as shown in Equation (8).

(8) $$P\left(A|B\right)=\frac{P\left(B|A\right)P(A)}{P(B)}$$

Furthermore, the final naive Bayesian distribution model can be obtained by calculating the posterior probability distribution, as shown in Equation (9).

(9) $$y=f(x)=argmax_{c_{k}}\frac{P(Y=c_{k})\prod_{j}P(X^{\left(j\right)}=x^{\left(j\right)}|Y=c_{k})}{\sum_{k}P(Y=c_{k})\prod_{j}P(X^{\left(j\right)}=x^{\left(j\right)}|Y=c_{k})}$$

The naive Bayes algorithm has a stable classification efficiency, can handle multiple classification tasks, is suitable for incremental training of small data, is relatively simple, and is suitable for text classification. However, due to the assumed prior model, the prediction performance is poor, and there is a certain error rate in classification decisions, which is sensitive to the expression form of input data.

#### 2.4.3. Support Vector Machine Algorithm

The support vector machine (SVM) algorithm is a classification algorithm that represents instances as points in space, separates data points with a straight line, and solves the optimal hyperplane by optimizing convex quadratic programming problems, which includes minimizing model complexity (minimizing the sum of squared weights) while limiting the misclassification of training samples. The SVM algorithm can map input features to high-dimensional space through the kernel function, making originally linear inseparable data linearly separable in high-dimensional space. Then, assume that the training set on a given feature space is illustrated in Equation (10), as follows:

(10) $$T=\left\{\left(x_{1},y_{1}\right),(x_{2},y_{2}),(x_{3},y_{3})\cdots (x_{N},y_{N})\right\}$$

where *x_i_* ∈ *R*, *y_i_* ∈ (−1, 1), *i* = 1, 2, 3~*N*, *x_i_* is the ith sample, and *y_i_* is the labeling of *x_i_*.

Based on the linearly separable training dataset proposed above, the separation hyperplane can be obtained by maximizing the interval, as described in Equation (11) [73]:

(11) $$y(x)=\omega^{T}\phi (x)+b$$

where *ϕ*(*x*) is a transformation function of some defined feature space that maps *x* to higher dimensions, i.e., the kernel function.

In addition, its corresponding classification decision function is given in Equation (12):

(12) $$f(x)=sign(\omega^{T}\phi (x)+b)$$

**Figure 9:**
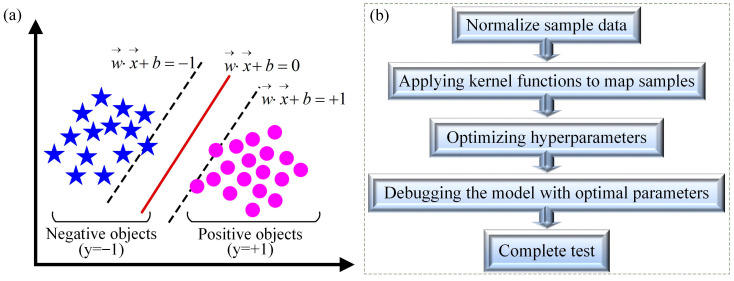
Schematic diagram of hyperplane in SVM algorithm (**a**) and modeling process (**b**).

Therefore, we need to find the hyperplane *y*(*x*) to optimally separate two sets. The schematic diagram of the optimal hyperplane is shown in Figure 9a.

The SVM algorithm can be used for classification, regression, and outlier detection, with good robustness. The process of the SVM model is shown in Figure 9b. The SVM algorithm is relatively efficient in high-dimensional space, but its explanatory power for high-dimensional mapping of kernel functions is not strong [74].

#### 2.4.4. Random Forest Algorithm

**Figure 10:**
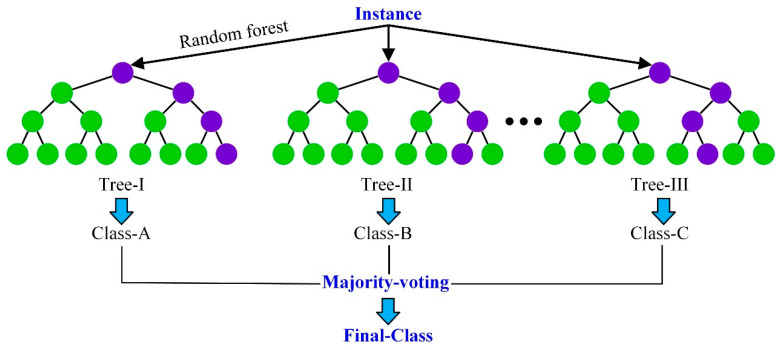
Process of random forest algorithm.

The random forest algorithm can be viewed as a set of decision trees, where each decision tree in the random forest estimates a classification, a process known as voting [75]. In an ideal scenario, we would choose the classification with the most votes based on each vote in each decision tree. In addition, in regression problems, the average of multiple regression results is taken as the final result. The specific process of the random forest algorithm is shown in Figure 10 [76].

The random forest algorithm can handle a large number of input variables and is able to assess the importance of variables when deciding on categories. In addition, for many kinds of information, the random forest algorithm can produce highly accurate classifiers that balance the error. However, the random forest algorithm loses the interpretability of the decision tree and may not improve the accuracy of the base learner in problems with multiple categorical variables.

#### 2.4.5. Artificial Neural Network Algorithm

**Figure 11:**
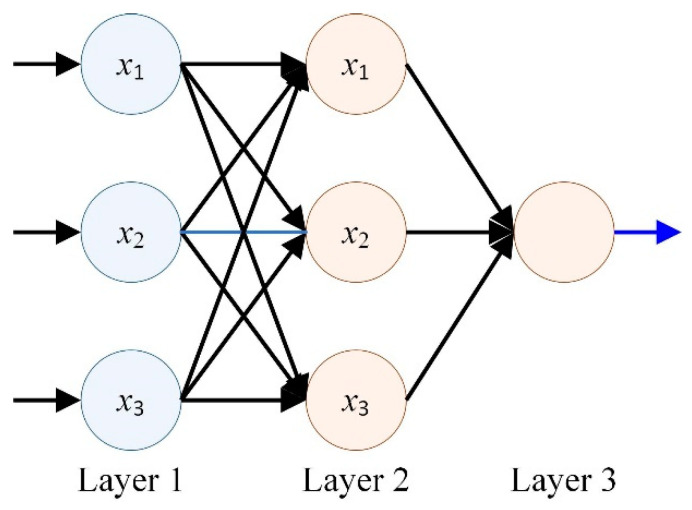
Schematic diagram of artificial neural network structure.

An abnormally complex network composed of artificial neural networks and neurons is generally similar, consisting of individual units connected to each other, with each unit having numerical inputs and outputs, which can take the form of real numbers or linear combination functions. The structure of the artificial neural network is shown in Figure 11. The artificial neural network is a widely parallel interconnected network composed of adaptive simple units, and its organizational structure can simulate the interaction reactions of biological neural systems to the real world.

Artificial neural network algorithms have a wide range of applications, including data analysis and mining, engineering pattern recognition, bioinformatics processing, and humanoid robots, among others [77]. For example, their applications include being virtual assistants (Siri, Alexa, etc.), traffic prediction (GPS navigation services), filtering spam and malware, rapid detection of cellular internal structures, deep space exploration, and so on [78,79].

On the other hand, adaptive Neuro Fuzzy Inference System (ANFIS) is a hybrid intelligent system that combines neural networks and fuzzy logic inference systems [80]. It utilizes the learning ability of neural networks to adjust the parameters of fuzzy logic systems, thereby optimizing fuzzy rules [81]. ANFIS is commonly used to handle modeling and control problems of complex systems with uncertainty and fuzziness. ANFIS is a Takagi Sugeno fuzzy model consisting of a series of If/Then rules, where the If part defines the fuzzy set and the Then part represents the output through linear or nonlinear functions [82]. ANFIS achieves parameter learning and optimization of these fuzzy rules through the structure of neural networks [83,84]. ANFIS can be applied in various scenarios in manufacturing processes, such as quality control (by analyzing data during the production process, predicting product quality, and conducting real-time monitoring and control during the production process) [85], fault diagnosis (analyzing equipment operation data can timely detect potential faults and abnormalities) [86], process optimization (optimize key parameters in the manufacturing process) [81,87].

Secondly, deep learning models include but are not limited to convolutional neural networks, recurrent neural networks, long short-term memory networks, and generative adversarial networks [88,89]. These models have achieved breakthrough results in fields such as image recognition, speech recognition, natural language processing, and gaming [90]. Deep learning can automatically extract features and process complex data. However, due to the high demand for data and intensive computing resources, training deep learning models requires a significant amount of computing resources [91,92]. In manufacturing processes, deep learning can be applied to various aspects such as quality inspection, predictive maintenance, process optimization, supply chain management, and more [93,94]. Overall, the application of deep learning in manufacturing processes can significantly improve the production efficiency, product quality, and the level of intelligence in supply chain management; however, at the same time, attention needs to be paid to its demand for data and computing resources, as well as the interpretability of models [95,96,97].

## 3. Optimization Objectives and Constraints of Process Parameters

### 3.1. Optimization Objective

**Figure 12:**
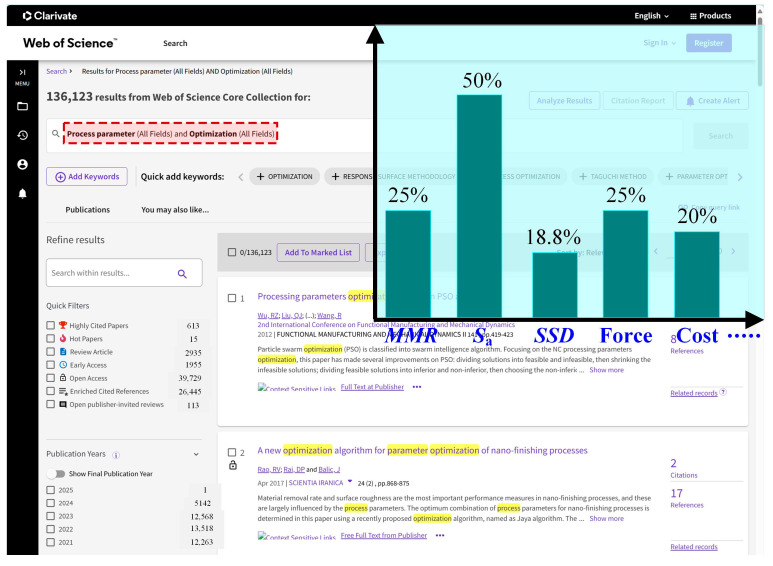
Typical types of optimization objectives reported in the literature.

Machining force, material removal rate, sub surface damage depth, residual stress, and residual strength are often optimization objectives that need to be considered in process parameter research. Reducing the machining force can suppress surface damage and improve machining surface quality; therefore, minimizing the grinding force is necessary. In addition, the material removal rate can reflect the processing efficiency of the actual production site of the parts, so it is necessary to maximize the material removal rate. Secondly, the service performance of the processed parts is affected by the sub surface depth, so it is necessary to reduce the sub surface damage depth to improve the machining quality of the parts and thereby increase the retention rate of material mechanical properties [98]. After searching in Web of Science using process parameter and optimization as keywords, 90 articles were randomly selected, and the types of optimization objectives were obtained, as shown in Figure 12.

According to Figure 12, it is found that most existing literature reports focus on optimizing the surface roughness and material removal rate, and the production cost of parts and sub surface damage of workpieces are also areas of concern for researchers. In addition, optimization objectives can generally be expressed using objective functions; common objective function expressions are shown in Equation (13) [78].

(13) $$\mathrm{minimize}\left(-f(MMR),f(S_{a})\cdots ,f(F_{n})\right)$$

### 3.2. Constraint Condition

Finding a set of parameter values under a series of constraint conditions to achieve the optimal objective value of a certain or a set of functions is a constraint problem in process parameter optimization [1]. The constraints can be either equality constraints or inequality constraints. The key to finding this set of parameter values is to meet the constraints and achieve the optimal target value. The constraint conditions are mostly within the parameter range set by the process experiment, and some also consider performance parameter requirements such as surface integrity of the workpiece [99].

## 4. Typical Applications of Optimization Algorithms in Practical Working Conditions

### 4.1. Genetic Algorithm

**Figure 13:**
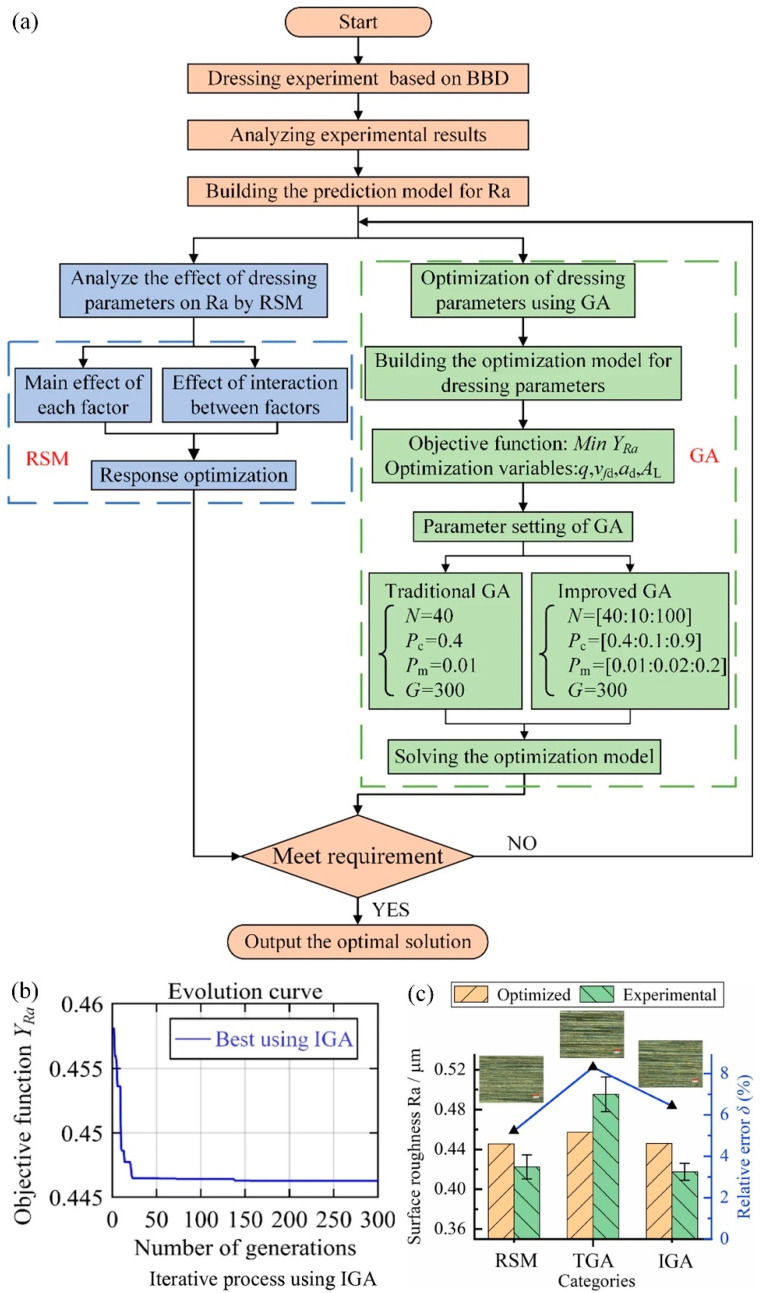
Optimization of process parameters in ultrasonic-assisted grinding based on improved genetic algorithm: (**a**) process parameter optimization process, (**b**) iterative optimization process, and (**c**) comparison of surface roughness before and after optimization.

This section mainly describes the relevant literature on using genetic algorithms to optimize process parameters, introduces the optimization objectives, constraints, and research results. Li et al. [100] established a mapping model between process parameters and surface roughness based on orthogonal experimental results, analyzed the influence weights of process parameters, and further established a process parameter optimization model using an improved genetic algorithm (as shown in Figure 13) to obtain the optimal process parameter solution for ultrasonic vibration-assisted grinding. Compared with the previous optimization, the surface quality was significantly improved, and the surface roughness was reduced. The results show that both the surface roughness prediction model and the process parameter optimization model based on the improved genetic algorithm have certain accuracy and reliability [101].

Optimizing the process parameters in ultrasonic vibration-assisted grinding is crucial for improving the surface integrity of the parts. In genetic algorithms, parameters such as population size *N*, crossover probability *P*, and mutation probability *P*_m_ are intervalized [100]. Then, use the improved genetic algorithm to find the optimal solution of the objective function. Develop a process parameter optimization model that incorporates constraint conditions and objective functions, as detailed in Equation (14). Calibrate the model by setting the iteration count and ensuring convergence (as depicted in Figure 13b). Subsequently, conduct a comparative analysis of the surface roughness outcomes and their residuals across various optimization strategies, as illustrated in Figure 13c.

(14) $$\left\{\begin{array}{c} MinY_{R_{a}}=Y_{R_{a}}(q,v,a_{p},A)\\ 0.2\leq q\leq 0.8\\ 50\leq v\leq 250\\ 20\leq a_{p}\leq 60\\ 0\leq A\leq 2.4 \end{array}\right.$$

Padhi et al. [102] used the genetic algorithm weighted sum method to search for the optimal machining parameter values, aiming to maximize the cutting efficiency and minimize surface roughness and dimensional deviation. They formulated the optimal search for machining parameter values as a multi-objective, multivariate, nonlinear optimization problem and evaluated its performance. Huang et al. [103] optimized the process parameters (laser power, cutting speed, auxiliary gas pressure, and focusing position) using a non-dominated sorting genetic algorithm (NSGAII) and outputted a complete optimal solution set, ultimately achieving nonlinear optimization of multi-objective parameters such as incision width, incision taper, and incision section roughness. Moreover, Zhao et al. [104] used the genetic algorithm (GA) to optimize the machining parameters of the NiTi shape memory alloy during dry turning in order to obtain better surface roughness and residual depth ratio. The optimized machining parameters were *v*_c_ = 126 m/min, *f* = 0.11 mm/rev, and *a_p_* = 0.14 mm. The ratio of surface roughness to residual depth was 0.489 μm and 64.13%, respectively.

In summary, genetic algorithms can be used to optimize various parameters, such as cutting parameters (cutting speed, feed rate, cutting depth, etc.), heat treatment parameters (temperature, time, cooling rate, etc.), and injection molding parameters (injection pressure, holding time, cooling time, etc.) [105,106]. By optimizing these parameters, product quality can be improved, production costs can be reduced, and production efficiency can be enhanced. The genetic algorithm is suitable for complex process parameter optimization problems [107]. However, in order to achieve optimal performance, careful selection and adjustment of algorithm parameters are required, and they may need to be combined with other optimization methods.

### 4.2. Response Surface Method

Response surface methodology is used in manufacturing process parameter optimization to determine the optimal combination of process parameters in order to improve product quality, reduce costs, and increase production efficiency [108]. Response surface methodology selects experimental design methods such as central composite design, Box–Behnken design, orthogonal design, etc., based on research objectives and parameter quantities. Some scholars use response surface methodology to optimize the detection of tool wear during machining, the optimization of machining trajectories for complex components, and process parameters [109,110]. In response surface methodology, it is necessary to consider experimental design, experimental factors and levels, select appropriate models, and then use model prediction and optimization algorithms (such as gradient descent, simplex method, etc.) to find the optimal combination of process parameters. Camposeco-Negrete [111] used central composite material design experiments and obtained regression models for energy consumption, specific energy, surface roughness, and material removal rate during the processing using response surface methodology. The sufficiency of the model was demonstrated through variance analysis, achieving the selection of process parameters for minimum specific energy consumption and minimum surface roughness [112]. Li et al. [100] designed an experiment on ultrasonic-assisted grinding and dressing of white alumina grinding wheels using the Box–Behnken method, established a predictive model for the surface roughness of bearing ring grinding, and analyzed the influence of process parameters (speed ratio, feed rate, dressing depth, and ultrasonic amplitude) on surface roughness using the response surface methodology [113]. It was found that speed ratio and ultrasonic amplitude are the main influencing factors of *R_a_*.

**Figure 14:**
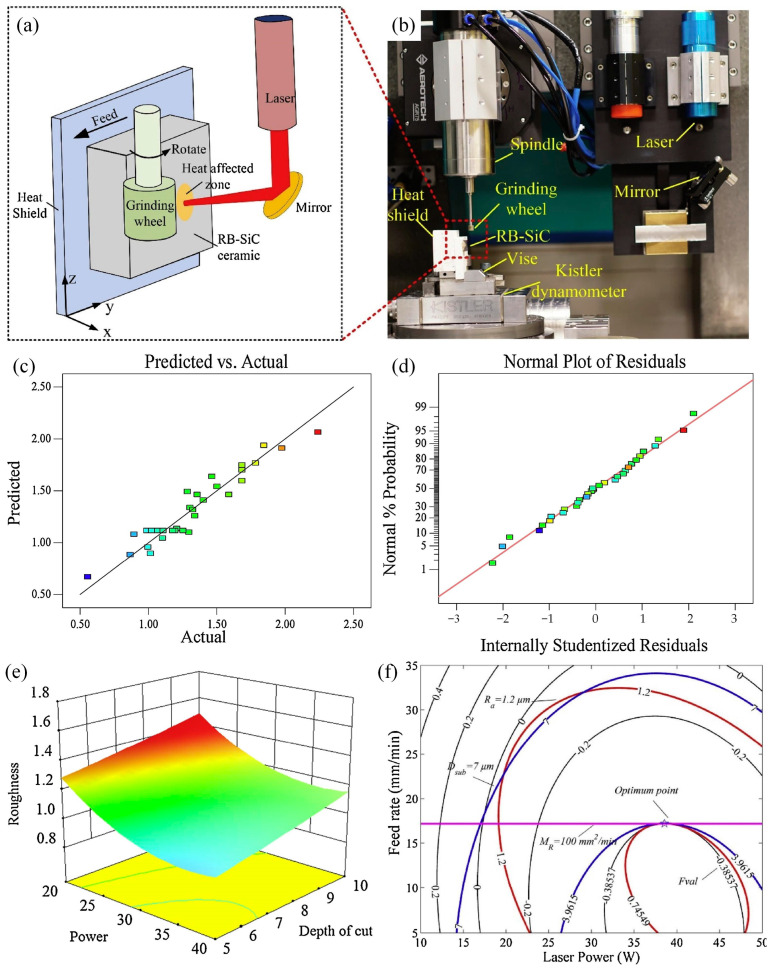
Optimization of RB SiC machining process with laser-assisted grinding: (**a**,**b**) schematic diagrams of the experimental system and the actual device, (**c**) predicted and actual response values of surface roughness, (**d**) probability distribution of standard residuals of surface roughness, (**e**) three-dimensional response surface diagram of surface roughness and process parameters, and (**f**) feasibility window of process parameters constructed through optimization, constraints, and objective functions.

However, response surface methodology combined with genetic algorithms is often used to optimize process parameters [114]. Response surface methodology is used to establish a predictive model for the objective function related to process parameters and experimentally verify the accuracy of the model. Then, genetic algorithms or improved genetic algorithms are used to find the optimal solution for the objective function [115]. Li et al. [116] used response surface methodology and genetic algorithm to optimize the process parameters in laser-assisted grinding RB-SiC machining. The schematic diagram and apparatus of the experimental system are shown in Figure 14a,b. Moreover, a four-factor and five-level process parameter experimental table was designed, as shown in Table 1, to achieve the minimum surface roughness, subsurface damage, and maximum material removal rate.

In addition, the genetic algorithm optimization method was used to optimize four types of process parameters: feed rate, spindle speed, laser power, and grinding depth. The objective function is shown in Equation (15), and the surface roughness empirical model, subsurface damage prediction model, and input variables were used as constraints to achieve maximum material removal efficiency. Further analyze the variance of surface roughness and the probability distribution of standard residuals and obtain a response surface graph between surface roughness and process parameters [117]. By applying the weighted sum method, the multi-objective optimization problem is simplified into a single-objective problem, and a Desirability function with the minimum normalization error is established, as shown in Equation (16). The contour map of the process parameters and the optimal solution of the calculation results are analyzed.

(15) $$Y=\phi \left(P,V,F,D\right)\pm \varepsilon$$

where *Y* is the response function and *ε* is the error.

(16) $$U\left(P,F,D,V\right)=W_{R_{a}}\left(\frac{R_{a}-{R_{a}}^{\prime }}{{R_{a}}^{\prime }}\right)+W_{D_{sub}}\left(\frac{D_{sub}-{D_{sub}}^{\prime }}{{D_{sub}}^{\prime }}\right)+W_{M_{R}}\left(\frac{{M_{R}}^{\prime }-M_{R}}{{M_{R}}^{\prime }}\right)$$

where *R_a_*′, *D_sub_*′, and *W_MR_*′ are constraints, and *R_a_*, *D_sub_*, and *W_MR_* are weighting factors.

### 4.3. Taguchi Method

The Taguchi method identifies and optimizes key factors affecting product performance through experimental design, while reducing process variability [118]. It particularly emphasizes reducing sensitivity to environmental changes and manufacturing process fluctuations in product design and manufacturing processes [119]. Some researchers focus on optimizing the process parameters of abrasive waterjet, laser drilling, dry cutting, and energy field-assisted mechanical manufacturing using the Taguchi method. They establish a mapping relationship between process parameters and processing quality, material removal rate, and tool wear; use residual distribution, regression equations, and experimental analysis to verify the accuracy of the prediction model; and obtain the optimal process parameters and influence weights [120,121]. Fan et al. [122] established an orthogonal experiment using the Taguchi method to analyze the influence of process parameters on the surface quality of microcrystalline glass optical free-form surfaces in laser-assisted rapid tool servo machining and established a mapping relationship between process parameters and surface integrity. Pradhan et al. [123] used the Taguchi method to analyze the relationship between material removal rate, tool wear rate, and machining process parameters during micro electrical discharge machining of mold steel and obtained the optimal electrical discharge machining process parameters (current, pulse time, and gap voltage).

**Figure 15:**
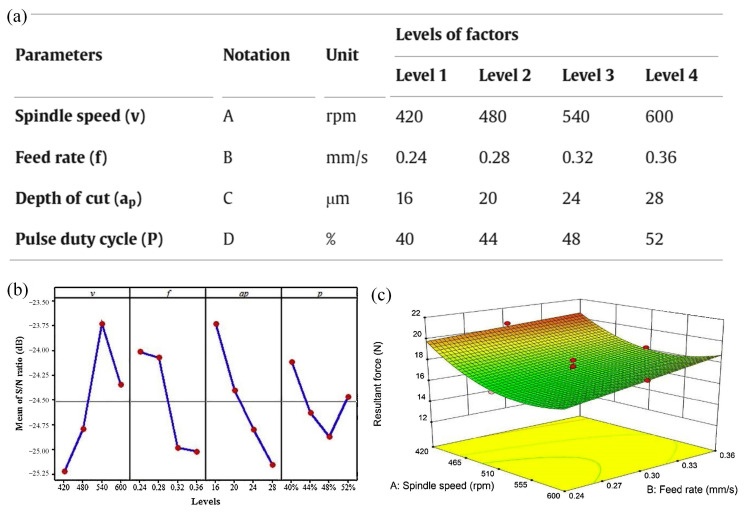
Optimization of process parameters for laser-assisted cutting based on Taguchi method: (**a**) parameter design of L16 orthogonal experiment, (**b**) average signal-to-noise ratio of cutting force, (**c**) response surface of the influence of spindle speed and feed rate on cutting force [125]. “Reprinted with permission from Ref. [125]. 2024, J. Manuf. Process”.

Moreover, K. Siva Prasad et al. [124] investigated the effects of water pressure, distance, abrasive flow rate, fiber orientation, material thickness, and abrasive particle size on surface quality during abrasive water jet machining (AWJM) of GFRP/epoxy composite materials. L_27_ orthogonal experiments were designed based on Taguchi’s method, and analysis of variance (ANOVA) was used to statistically analyze the experimental results. The AWJM process parameters and response parameters were correlated, and the experimental results showed that abrasive particle size is the main factor affecting surface roughness. Song et al. [125] studied the influence of spindle speed, feed speed, cutting depth, and laser pulse duty ratio on the cutting force in the process of fused silica LAM based on the Taguchi method (TM) experiment (as shown in Figure 15). An orthogonal experiment and central composite design experiment were used to analyze the square error (ANOVA), signal-to-noise ratio (*S*/*N*), main effect diagram, three-dimensional response surface, and its corresponding contour map to evaluate the influence of various factors on the cutting force [126,127,128].

It should be noted that the reference [20]’s definition of the signal-to-noise ratio is shown in Equation (17).

(17) $$S/N=-10\log[\frac{1}{n}\left({y_{1}}^{2}+{y_{2}}^{2}+\cdots +{y_{n}}^{2}\right)]$$

In the formula, *S*/*N* represents the response value, while *y*_1_, *y*_2_~*y_n_* represent the output values under *n* repeated test conditions [129].

Suleyman Simsek et al. [130] designed three levels as control parameters using the Taguchi method and L_27_ orthogonal experiment and optimized them to obtain the optimal combination of control parameters to optimize response characteristics. The maximum error between the optimization results and experimental results was 9.42%. Carmita Camposeco Negrete [111] analyzed the effects of cutting depth, feed rate, and cutting speed on response variables using orthogonal experiments, signal-to-noise ratio (*S*/*N*), and analysis of variance (ANOVA), and introduced the Taguchi method to identify the main effects. In addition, many researchers have combined the Taguchi method with optimization algorithms such as the genetic algorithm and response surface methodology to develop prediction models for surface roughness, cutting force, subsurface damage, or other objectives [131]. They have also used Design Expert 13 and Minitab software 21.1 to estimate and analyze the significance of the regression models and finally obtained the optimal process parameters to analyze surface integrity. Li et al. [11] proposed a combination of the Taguchi method, response surface method, and NSGA-II method to optimize the injection molding process of fiber-reinforced composite materials. Based on orthogonal experimental design, the importance of various parameters on the three quality objectives was studied through analysis of variance (ANOVA). Three response surface models were created to map the complex nonlinear relationship between design parameters and quality objectives. Finally, genetic algorithm II (NSGA-II) was connected to the prediction model to find the optimal design parameter value.

### 4.4. Particle Swarm Optimization

**Figure 16:**
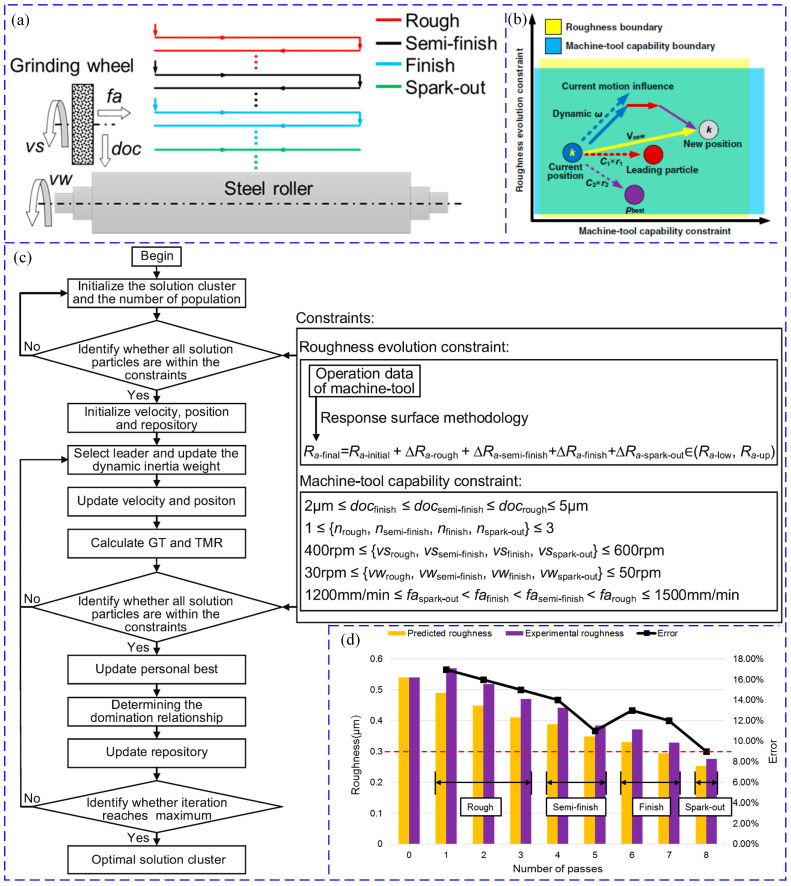
Optimization study of process parameters for multi-pass roller grinding: (**a**) schematic of multi-pass roller grinding, (**b**) solution of particle position update program, (**c**) hybrid particle swarm optimization algorithm flow, and (**d**) changes in surface roughness prediction and actual values.

The particle swarm optimization algorithm simulates the foraging behavior of bird flocks and seeks the optimal solution through collaboration and information sharing among individuals in the group [132]. Particle swarm optimization can achieve multi-objective optimization and optimize multiple objectives simultaneously. Additionally, it can be combined with other optimization techniques such as genetic algorithms and simulated annealing to improve optimization performance [132,133]. The application cases of particle swarm optimization in practical manufacturing process optimization are increasing, such as successful applications in mechanical processing, material processing, chemical process optimization, and other fields [134]. Latchoumi et al. [132] used particle swarm optimization (PSO) technology to solve water jet machining related to cavitation shot peening problems. Group initialization starts from water pressure, standing distance, and lateral velocity, and the fitness estimation of PSO is residual stress, hardness, and surface contour roughness. These related and independent parameters are used for water jet machining to induce beneficial residual stresses in the surface layer. Chen et al. [4] proposed a multi-objective optimization process method for mixed particle swarm optimization of multi-pass roller grinding (the principle of multi-pass roller grinding is shown in Figure 16a), combined with a response surface model for surface roughness evolution. Hybrid particle swarm optimization considers the entire grinding process parameters as a whole and achieves parameter optimization by considering multi-objective and constraint conditions. A response surface model for surface roughness evolution was established based on process parameters such as rough grinding, semi precision grinding, and precision grinding [98]. The effectiveness of the mixed particle swarm multi-objective optimization method was verified through experiments (Figure 16c). The results showed that compared with experimental roughness, the error between predicted roughness and experimental roughness was less than 16.53%, and the grinding efficiency was improved by 17.00% (Figure 16d).

Li et al. [28] proposed a complex cutting parameter optimization method based on the Taguchi method, response surface methodology (RSM), and multi-objective particle swarm optimization algorithm (MOPSO), with energy efficiency and processing time as the objectives. A response regression model was established using RSM, and the process parameters with the smallest specific energy consumption and processing time were determined using an improved MOPSO algorithm. Finally, a balance value was obtained between processing time and energy consumption. In the process of optimizing process parameters, the particle swarm optimization algorithm can consider a parameter adjustment strategy, improve local search ability, and prevent the premature convergence of particle swarm to non-optimal solutions by introducing a diversity preservation mechanism or penalty mechanism [135]. In addition, it can develop effective strategies to address constraints in optimization problems, such as upper and lower limits of process parameters, process stability, etc. Of course, it can also improve the algorithm’s adaptability to different problems and problems of different scales [136].

### 4.5. Other Algorithms

**Figure 17:**
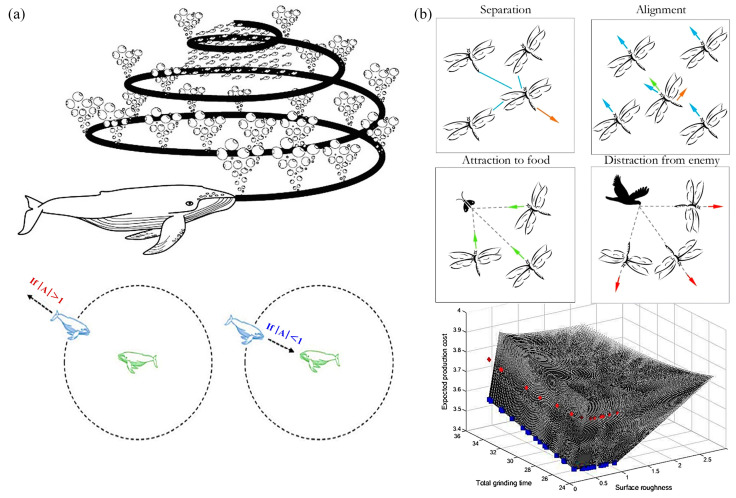
Applications of other optimization algorithms: (**a**) sine cosine whale optimization algorithm; (**b**) multi-objective dragonfly algorithm and its feasible trade-off solution set [114,137]. Reprinted with permission from Refs. [114,137]. 2024, Neural Comput. Appl and *J. Ind. Prod. Eng*”.

Soheyl Khalilpourazari et al. [137] proposed a sine cosine whale optimization algorithm, as shown in Figure 17a, to minimize the total production time in the multi-pass milling process. Zheng et al. [138] combined the update operator of the sine cosine algorithm with the whale optimization algorithm and studied the performance of three optimization algorithms in experimental studies.

In addition, Guo et al. [139] used the multi-objective particle swarm optimization algorithm to optimize the prediction model of surface roughness and cutting force based on crowded distance sorting and determined the optimal combination of process parameters to reduce surface roughness and achieve a smaller cutting force. Pramanik et al. [140] studied the influence of laser beam processing parameters on the cutting of titanium high-temperature alloys and applied particle swarm optimization technology based on the metaheuristic algorithm to model and optimize the quality characteristics in laser cutting. Soheyl Khalilpourazari et al. [114] aimed to optimize the grinding process parameters while optimizing the surface quality, grinding cost, and total processing time of the parts. They adopted a multi-objective dragonfly algorithm (as shown in Figure 17b) and used a weighting method to optimize the three objectives into one. The research results showed that the proposed optimization algorithm has significant advantages compared to traditional genetic algorithms [141]. Mohamed Arezki Mellal et al. [18] used the rhododendron optimization algorithm to minimize the total production time in multi-pass milling processes, successfully addressed constraints, and compared the optimal results with those of other optimization algorithms. Lin et al. [142] used the Convolutional Neural Fuzzy Network (1DCNFN) to establish a surface roughness prediction model and then used the particle swarm optimization algorithm to optimize the process parameters in an ultrasonic-assisted grinding process. Li et al. [16] used the black hole continuous ant colony algorithm to optimize the grinding specific energy and surface roughness of CNC machine tools during the grinding process and solved the optimal combination of ant colony algorithms. Finally, they found that this algorithm can effectively solve the optimization problem that traditional algorithms are prone to getting stuck on in local areas.

## 5. Conclusions

This article systematically reviews the application and development trends of optimization algorithms in practical engineering production. It explores the classification, definition, and research status of process parameter optimization algorithms in engineering manufacturing processes, both domestically and internationally. Furthermore, it provides a detailed introduction to the specific applications of various optimization algorithms in actual working conditions. Based on this analysis, the following conclusions regarding the current development status of process parameters in engineering applications using optimization algorithms were drawn.

(1) Currently, the response surface method, genetic algorithm, Taguchi method, and particle swarm optimization algorithm are relatively mature regarding their stage of development and have been extensively applied in process parameter optimization. These methods are increasingly utilized as optimization targets in various aspects of manufacturing, including surface roughness, subsurface damage, cutting force, and mechanical properties, whether in mechanical processing or special processing. However, these optimization algorithms have reached a level of maturity and are no longer novel.
(2) In addition, as existing optimization algorithms continue to evolve and be updated, machine learning and other advanced optimization algorithms will emerge. When establishing mathematical optimization models, these approaches consider specific problem-specific analyses and set objective functions and constraints for optimization objectives.
(3) However, the constraints encountered in real-world engineering manufacturing processes are frequently intricate and often entail multi-objective optimization challenges, where distinct optimization goals are inter-related by specific constraints. This intricate nature poses a significant obstacle to the formulation of an optimization model that can effectively address multiple objectives at once. Subsequent research has demonstrated that employing a weighting method can effectively consolidate multiple objectives into a unified target, thereby facilitating the identification of optimal process parameter solutions.
(4) With the advent of intelligent manufacturing, the application of optimization algorithms in machining technology increasingly relies on data-driven and real-time feedback mechanisms. This trend enhances the intelligence and automation of the production process. These optimization algorithms not only bolster the competitiveness of the manufacturing industry but also drive the evolution of manufacturing technology towards greater efficiency, sustainability, and customization.

## 6. The Development Trend of Optimization Algorithms

**Figure 18:**
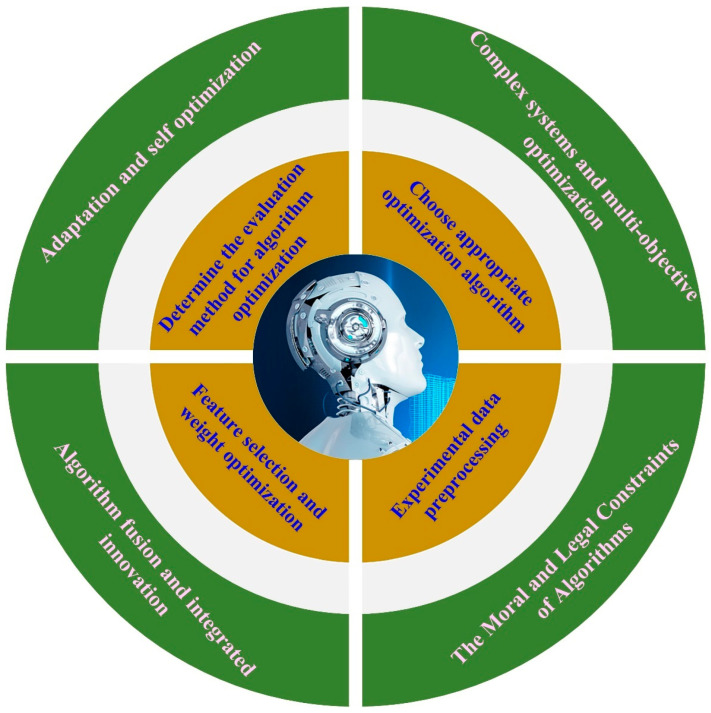
Future research directions for optimization algorithms.

The aforementioned analysis suggests that in the future, intelligent manufacturing processes will predominantly focus on technologies such as deep reinforcement learning and digital twinning for process optimization. Process optimization algorithms represent an effective technical approach to achieving intelligent manufacturing. These algorithms can substantially enhance the manufacturing efficiency, decrease production costs, elevate the quality of components, and provide new technical support for the optimization of process parameters. Looking ahead, the following areas are poised to become the primary research focal points, as depicted in Figure 18.

(1) Adaptation and self-optimization

In the future, optimization algorithms need to be designed with stronger capabilities and better performance for adaptive and self-adjusting algorithms. In actual production and manufacturing, process parameter optimization can be achieved by self-adjusting and dynamically adjusting parameters, reducing manual intervention. Moreover, advanced optimization algorithms will be capable of performing online adjustments to model parameters or adaptively optimizing the model structure.

(2) Complex systems and multi-objective optimization

The challenges of practical engineering applications are notably complex. Consequently, future optimization algorithms must concentrate more on multi-objective optimization processes, multi-constraint interactions, and high-precision function design to effectively search for optimal solutions across multiple objectives. The optimization of process parameters ultimately aims to enhance production quality, reduce waste and costs, and meet production goals such as low-carbon environmental protection and green manufacturing. Therefore, complex systems and multi-objective optimization in production can be systematically addressed in the future.

(3) Algorithm fusion and integrated innovation

Future optimization algorithms could consider the integration and fusion of multiple algorithms (including deep learning, particle swarm optimization, genetic algorithms, and other intelligent optimization techniques) to create a more efficient, flexible, intelligent, and high-performance hybrid optimization strategy. This strategy can not only be utilized to find certain engineering parameters but can also be applicable to a range of other optimization challenges and engineering domains.

## Figures and Tables

**Table 1:** Level of independent factors.

Factor	Factor Level				
	−2	−1	0	1	2
Power *P* (W)	10	20	30	40	50
Speed *V* (rpm)	6000	8000	10,000	12,000	14,000
Feed rate *F* (mm/min)	5	15	25	35	45
Grinding depth *D* (μm)	2.5	5	7.5	10	12.5

## Data Availability

No new data were created or analyzed in this study.
